# Oxygen generating biomaterials at the forefront of regenerative medicine: advances in bone regeneration

**DOI:** 10.3389/fbioe.2024.1292171

**Published:** 2024-01-12

**Authors:** Jiayi Zhao, Chao Zhou, Yang Xiao, Kunyan Zhang, Qiang Zhang, Linying Xia, Bo Jiang, Chanyi Jiang, Wenyi Ming, Hengjian Zhang, Hengguo Long, Wenqing Liang

**Affiliations:** ^1^ Department of Orthopaedics, Zhoushan Hospital of Traditional Chinese Medicine Affiliated to Zhejiang Chinese Medical University, Zhoushan, China; ^2^ Department of Orthopedics, Zhoushan Guanghua Hospital, Zhoushan, China; ^3^ Medical Research Center, Zhoushan Hospital of Traditional Chinese Medicine Affiliated to Zhejiang Chinese Medical University, Zhoushan, China; ^4^ Rehabilitation Department, Zhoushan Hospital of Traditional Chinese Medicine Affiliated to Zhejiang Chinese Medical University, Zhoushan, China; ^5^ Department of Pharmacy, Zhoushan Hospital of Traditional Chinese Medicine Affiliated to Zhejiang Chinese Medical University, Zhoushan, China

**Keywords:** bone defects, tissue engineering, bone regeneration, controlled oxygen-releasing biomaterial, regenerative therapy

## Abstract

Globally, an annual count of more than two million bone transplants is conducted, with conventional treatments, including metallic implants and bone grafts, exhibiting certain limitations. In recent years, there have been significant advancements in the field of bone regeneration. Oxygen tension regulates cellular behavior, which in turn affects tissue regeneration through metabolic programming. Biomaterials with oxygen release capabilities enhance therapeutic effectiveness and reduce tissue damage from hypoxia. However, precise control over oxygen release is a significant technical challenge, despite its potential to support cellular viability and differentiation. The matrices often used to repair large-size bone defects do not supply enough oxygen to the stem cells being used in the regeneration process. Hypoxia-induced necrosis primarily occurs in the central regions of large matrices due to inadequate provision of oxygen and nutrients by the surrounding vasculature of the host tissues. Oxygen generating biomaterials (OGBs) are becoming increasingly significant in enhancing our capacity to facilitate the bone regeneration, thereby addressing the challenges posed by hypoxia or inadequate vascularization. Herein, we discussed the key role of oxygen in bone regeneration, various oxygen source materials and their mechanism of oxygen release, the fabrication techniques employed for oxygen-releasing matrices, and novel emerging approaches for oxygen delivery that hold promise for their potential application in the field of bone regeneration.

## 1 Introduction

Tissue defects may arise as a consequence of either congenital anomalies or acquired pathological conditions. The latter encompasses the occurrence of tissue damage resulting from various aetiologies, such as pathological processes, traumatic events, or surgical interventions (Bara et al., 2018). Numerous methodologies have been devised for the management of tissue defects, encompassing the utilization of autografts, allografts, and xenografts. While autografts have long been the preferred choice, their procurement results in the co-occurrence of comorbidity at the donor site, and the availability of autografts is also limited (Ansari et al., 2022). One of the primary concerns inherent in the utilization of grafts pertains to the post-transplant viability of cells (Grounds, 2018). The utilization of allografts was investigated, particularly in instances involving osseous and cutaneous tissues (Mamidi et al., 2022). The utilization of xenografts has been proposed as an alternative approach; however, it is imperative to note that this course of action may elicit significant immune responses and potentially entail the transmission of infectious agents (Fernandes et al., 2022). Furthermore, the utilization of animal-derived sources as potential alternatives is impeded by ethical and cultural considerations. The ongoing pursuit of alternatives has encompassed the utilization of alloplastic materials for the purpose of regenerative application as well as the exploration of cellular replacement therapy, specifically involving hormone-secreting cells. Additionally, the investigation has extended to the examination of biomolecules that hold potential for facilitating tissue regeneration, particularly within the skeletal system and the integumentary system (Margiana et al., 2022; Samandari et al., 2022).

In an alternative perspective, there have been suggestions for the utilization of tissue engineering methodologies that involve the amalgamation of biomaterials with cellular components. In recent times, the utilization of the three-dimensional (3D) bioprinting technique has been employed for the purpose of cultivating living tissue constructs in controlled laboratory environments. Additionally, a multitude of bioinks have been formulated to facilitate the construction of 3D biomimicking models, thereby encompassing the intricate characteristics of tissues in relation to their biological, physical, and mechanical properties (Ashammakhi et al., 2019a). Due to the fact that the presence of functional cells within a bone construct is crucial for tissue-engineered bone to effectively repair large bone defects, scientists evaluated the functionality of mesenchymal stem cells (MSCs) subsequent to an extended period of continuous severe hypoxia (Deschepper et al., 2011). Consequently, it is important for newly developed capillaries (a process known as angiogenesis) to be promptly established within the transplanted tissues in order to address the limitations of oxygen diffusion. Given that angiogenesis requires a certain amount of time to occur, it is vital to develop a strategic approach to address this temporal gap. This approach is necessary to maintain a continuous and uninterrupted provision of essential nutrients and oxygen to implanted constructs, thereby preventing the untimely demise of cells (Coronel et al., 2019).

The oxygen supply during graft integration is crucial to the success of engineered bone in regenerative engineering. Tissue necrosis and programmed cell death occur due to a lack of oxygen (Hirao et al., 2007). Blood is the main transport medium for oxygen and nutrients throughout the body’s vascular system. The diffusion limit of oxygen and nutrients within a tissue is considered to be 200 μm from a vessel. Therefore, all cells must be within 200 μm of a vessel in order for the engineered tissue to be sustainable and for optimal vasculature to supply sufficient nutrients. Postimplantation complete vascularization typically necessitates a gradual progression (Rouwkema and Khademhosseini, 2016). The process of achieving 83% vascularity in a transplant may take approximately 6 weeks. This period allows for the integration of the host’s capillaries and blood arteries into the designed implant (Rouwkema and Khademhosseini, 2016).

In a broad sense, oxygen can be administered through direct means, such as utilizing perfluorocarbon-based systems that release oxygen, or alternatively, it can be conveyed through the use of a carrier composed of biomaterials (Corrales-Orovio et al., 2023). Various biomaterials have been extensively investigated as scaffolds; however, the incorporation of oxygen-releasing agents remains a relatively unexplored area (Liang et al., 2021). A major issue that has arisen pertains to the rapid release of oxygen, which has the potential to exhibit cytotoxic effects on cells. The provision of sustainable oxygen release can offer support to cells existing within the implanted construct prior to the occurrence of angiogenesis, which typically takes place within a period of one to 2 weeks. Once angiogenesis occurs, new capillaries take over the responsibility of supplying oxygen to the cells (Ashammakhi et al., 2019b). This problem has been predominantly resolved through the use of hydrophobic carrier biomaterials, which possess the ability to gradually release oxygen over extended durations, lasting up to a maximum of 10 days (White et al., 2014). The *in vitro* experiments have established the efficacy of these biomaterials and their effect on cell viability. Moreover, apart from providing assistance to developed constructs during the crucial period immediately following implantation, materials that generate oxygen can also be beneficial in the management of injured tissues, such as persistent wounds and complications arising from the obstruction of blood vessels that supply nutrients, for example, myocardial infarction (Fan et al., 2018). In addition, materials capable of generating oxygen can be employed to provide support for cells with higher metabolic activity, including neurons, hepatocytes, and muscle cells. To date, the majority of research work has been directed towards the combining of diverse carrier biomaterials with an oxygen source. The literature has shown that there is evidence of advantageous outcomes in the fields of bone and muscular tissue engineering (Touri et al., 2018; Agarwal et al., 2021).

This review focuses on the examination of oxygen source materials, carrier scaffolds, production techniques, release mechanisms, characterization methodologies, and their impact on cellular behavior and *in vivo* experimentation. In addition, we emphasize the difficulties and prospects and provide a concise overview of recent advancements in this dynamic and significant field. Anticipated are further advancements and implementations of oxygen generating systems, which are projected to significantly impact the future of engineered tissue structures and their clinical applications.

## 2 Challenges and oxygen’s crucial role in tissue regeneration

The optimal bone-engineered scaffolds commonly encompass the essential constituents that promote cellular viability and proliferation, facilitating the development of the bone (Abdollahiyan et al., 2021). In the field of bone regeneration, the vital role of nutrients and growth factors cannot be overstated. Similarly, the provision of an optimal oxygen supply is an imperative requirement. Oxygen, a vital element, plays a crucial role in sustaining cellular viability within scaffolds. The distribution of oxygen within the sophisticated scaffolds of 3D structures poses a formidable challenge in the field of bone engineering. In the case of relatively delicate structures, such as scaffolds measuring 1-2 mm in thickness, one can enhance the presence of oxygen by permitting the gentle movement of fluid carrying dissolved oxygen to permeate through the interconnecting pores of said scaffold. However, the development of porous scaffolds may potentially compromise their mechanical strength, particularly in scenarios where stiff or rigid scaffolds are necessary (Duan et al., 2022). This obstacle can greatly hinder the advancement of bone-engineered constructs from the laboratory to practical applications in healthcare. In order to confront the obstacles associated with hypoxia in expansive structures, it is possible to develop scaffolds that possess connected vascular mimetic channels, thereby enabling the perfusion of media (Chen et al., 2021). However, the construction of capillaries and the establishment of resilient vascular structures within the scaffolds continue to present a significant challenge (Shahabipour et al., 2020). A significant limitation within the discipline of bone engineering is the slow process of vascularization observed in implanted engineered scaffolds (Guo et al., 2022). The enhancement of vascularization, through the implementation of techniques such as the utilization of gene delivery, nanomaterials, and angiogenic molecules, holds the potential to amplify vascularization to a certain degree.

To address the major challenges associated with cell viability, researchers have resorted to employing cell-free, biologically active scaffolds as an *in-situ* technique for bone engineering (Augustine et al., 2018). However, to achieve favorable outcomes in the context of an *in-situ* transplantation strategy, it is important that the cells residing within the adjacent tissue exhibit migratory capabilities towards the designated implantation site, coupled with a suitable rate of cellular proliferation. This concerted effort is required for the purpose of effectively restoring and rejuvenating the impaired bone (Park et al., 2021). In contrast to the cell-laden scaffolds, the requirement for prevascularization or rapid vascularization of the structures upon insertion is not essential in this particular technique. However, it is necessary to have both simultaneous vascularization and cell migration in order to mitigate the occurrence of cell death induced by hypoxia. Although the use of nanomaterials and angiogenic growth factors shows promise, it is important to note that angiogenesis does not occur immediately and does not align with the proliferation and migration rates of other cells (Wen et al., 2023). Therefore, the incorporation of supplementary oxygen sources within 3D scaffolds has the potential to improve cellular viability and facilitate cellular proliferation until the formation of a fully functional vasculature.

Despite the potential of 3D bioprinting as a viable method for producing substantial tissue structures, its use in clinical settings is constrained by different factors. The diffusion of oxygen and media within the construct, as well as the elimination of metabolic waste from the bioprinted thick structures, pose significant challenges. In the pursuit of fabricating constructs with interconnecting pores, modern day bioprinting methodologies incorporate the use of pliable hydrogels within bioinks to facilitate optimal diffusion of oxygen and media within the constructs. However, within the scope of mechanically firm bioprinted constructs, one has to recognize the potential compromise in the diffusion of oxygen and media throughout the entirety of said construct. In addition, the task of developing connected microscale porosity to enhance the diffusion of media presents an enormous obstacle, primarily resulting from the constrained resolution of conventional printing methods (Erdem et al., 2020). Furthermore, the dearth of oxygen following the introduction of the 3D bioprinted constructs may lead to cellular death and, ultimately, the downfall of the implanted structure. Thus, the integration of oxygen-generating substances within bioprinted constructs presents a viable solution to address the issues associated with hypoxia across various stages, including the printing procedure, *in vitro* development, and implantation phases.

## 3 Oxygen generating biomaterials (OGBs)

Researchers have diligently examined novel OGBs with the aim of demonstrating their clinical implications. These investigations have been focused on determining the potential of OGBs to facilitate sustained and controlled oxygen release, thereby enhancing cellular survival and optimizing cellular function (Sun et al., 2022). Previous research has demonstrated that various chemical compounds, such as hydrogen peroxide (H_2_O_2_), sodium percarbonate (SPO), calcium peroxide (CPO), magnesium peroxide (MPO), and fluorinated compounds, specifically perfluoromethyl-cyclohexyl piperidine, possess the capability to produce or supply oxygen within artificially designed structures, with the purpose of facilitating the process of tissue regeneration or repair (Suvarnapathaki et al., 2019; Guo et al., 2020; Draper et al., 2022; Samanipour et al., 2023).

The oxygen release rate is a critical factor in the formation of tissue constructs. For instance, in the event of an excessively abrupt oxygen release, the oxygen becomes ineffective as a result of supersaturation. Conversely, inadequate oxygen release results in an insufficient supply of the elemental energy required to sustain optimal cellular performance. Therefore, the potential for controlled and continuous oxygen supply may have significant consequences for biological systems. Temperature, pH, the solid peroxide-to-water ratio, the amount of catalyst, and the type of catalyst are all important in determining the oxygen production rate from peroxide compounds (Soleymani et al., 2011). The rate at which oxygen is released from its source is affected not only by its own hydrophobicity, but also by that of the surrounding biopolymer. If, for instance, solid peroxides are encased in a hydrophobic substance, the passage of water into the hydrophobic material slows down the oxygen release reaction (Pedraza et al., 2012). In this situation, solid peroxide particles are not rapidly immersed in water, resulting in a gradual release of oxygen. However, solid peroxide particles begin degrading and producing oxygen more quickly in the presence of hydrophilic materials due to the rapid adsorption of water. Oxygen-releasing biomaterials, both hydrophilic and hydrophobic, have been employed in a variety of tissue engineering applications (Harrison et al., 2007; Oh et al., 2009; Fraker et al., 2011). Since the rate of O_2_ release from H_2_O_2_ breakdown was shown to increase dramatically with increasing H_2_O_2_ concentration, it was concluded that the disproportionation of H_2_O_2_ to O_2_ followed first-order kinetics (Harrison et al., 2007). Sudden oxygen release is a typical issue with oxygen delivery materials and can be harmful to cells. Furthermore, increased production of reactive oxygen species like H_2_O_2_ may cause adverse side effects. Extracellular ROS oxidize lipoproteins and trigger collagen-degrading matrix metalloproteinases, whereas intracellular ROS can react frequently with a variety of substances including proteins, lipids, carbohydrates, and nucleic acids (Eks et al., 2015). ROS (reactive oxygen species) products initiate multiple chain reactions that result in the generation of further radicals. These radicals are more detrimental than their predecessors, hence facilitating the propagation of damage throughout the bone tissue (Betteridge, 2000). Therefore, it is crucial to develop biomaterials with the ability to release oxygen continuously for use in tissue engineering. To allow for revascularization and maturity of the designed graft inside the host system, the release kinetics of an ideal oxygen-releasing biopolymer should be adjustable and extend from days to weeks (Pedraza et al., 2012).

### 3.1 Oxygen source materials

Therapeutic oxygen can be administered in various forms, including gas, liquid, or solid sources. Hyperbaric oxygen has been used for the management of skin wounds in its gaseous form. However, this particular methodology presents various complexities, including the necessity for repeated treatments and pulmonary injury (Ortega et al., 2021). In order to achieve sustained and extended delivery, an investigation was carried out to explore the use of both liquid and solid sources of oxygen. The sources of oxygen currently use include H_2_O_2_, SPO, CPO, and MPO. H_2_O_2_, a liquid with a boiling point of 150.2°C, spontaneously decomposes to produce water and oxygen through a disproportionation reaction. In clinical practice, a solution containing 3% H_2_O_2_ is frequently utilized. However, it is important to note that the impact of H_2_O_2_ on cells is reliant upon the dosage administered (Xiang et al., 2016). At lower concentrations, hydrogen peroxide (H_2_O_2_) has been observed to facilitate cellular proliferation. However, when the concentration reaches 0.4 mM, it functions as a potent inducer of apoptosis across a wide range of cell types.

Solid peroxides go through initial dissociation, yielding H_2_O_2_, which subsequently undergoes decomposition into water and oxygen. The kinetics of oxygen release from peroxide compounds can be influenced by various factors, including temperature, pH, and the presence of catalase (Wang et al., 2016). Moreover, the decomposition and purity of solid peroxides play a significant role in determining the rate at which oxygen is released from their carriers (Zhou et al., 2023). Just like H_2_O_2_, SPO exhibits solubility in water and facilitates the immediate release of oxygen. The slow oxygen release kinetics of MPO can be attributed to its relatively low decomposition rate. The commercially available MPO exhibits a purity range of 15–25%, whereas the CPO demonstrates a higher purity range of 60–80% (Cassidy and Irvine, 1999). In the context of SPO (20%–30% purity), the availability of H_2_O_2_ ranges from approximately 0.25–0.4 mol. CPO is widely acknowledged within the scientific community as a peroxide compound with a high degree of safety. Its utilization has been favored in the development of biomaterials that generate oxygen (Camci Unal et al., 2013). One of the main challenges encountered when employing solid peroxides as chemical sources of oxygen is the harmful side effects resulting from byproducts like ROS, H_2_O_2_, higher pH levels, and metal ions (Wang et al., 2011).

Perfluorocarbons (PFCs) are hydrolipophilic, chemically inert, biocompatible hydrocarbons in which the hydrogen atom is fluorine. These PFCs possess minimal polarizability, which renders them exceptionally well-suited for biological applications as oxygen carriers (Riess, 2005; Jägers et al., 2021). By means of van der Waals forces, PFCs physically entangle the molecules of O_2_, thereby dissolving it. The oxygen solubility coefficient of PFCs is significantly greater, approximately 20 times higher, in comparison to that of blood (Lowe et al., 1998). According to Henry’s law, it correlates linearly with the ambient O_2_ partial pressure. That’s why PFCs can store and release oxygen according to the metabolic needs and oxygen saturation level of a certain tissue. Moreover, PFCs have demonstrated their suitability as viable options for bone regeneration. It has been demonstrated that implanting a PFC nanoemulsion increases the concentration of oxygen at the site of bone fractures and accelerates bone regeneration in rabbits with fractures that were studied under hypoxic conditions. Consequently, PFCs boosted levels of beneficial cytokines such vascular endothelial growth factor (VEGF) and matrix metalloproteinase 9 (MMP-9) and encouraged osteoblast development (Wang et al., 2020a).

Oxygen can be introduced into microtanks under hyperbaric conditions. After being incorporated into a secondary biomaterial, oxygen has the ability to diffuse out of it in a manner determined by the thickness and permeability of the microtank shell. In clinical settings, oxygen microbubbles have found widespread application, particularly as an ultrasound contrasting agent. These microbubbles assume the form of liquid foams that are stabilized by surfactants, lipids, or various polymers. Significantly, ultrasounds *in vivo* have the capability to rupture oxygen bubbles locally, which presents a promising characteristic for the treatment of hypoxic sites (Eisenbrey et al., 2015).

Photosynthetic algae are another source for molecular oxygen. Numerous photosynthetic pigments, including chlorophyll, are abundant in algae, a diverse group of photosynthetic organisms that can convert carbon dioxide into oxygen under light stimulus in a sustainable manner. Photosynthetic algae as oxygen-releasing materials represent an attractive option and have been explored both *in vitro* and *in vivo* for tissue engineering and regeneration due to the existence of algae’s symbiotic relationship with a wide range of eukaryotic hosts, including animals (Maharjan et al., 2022).

### 3.2 Oxygen carrier biomaterials

The utilization of biomaterials as carriers for oxygen sources is necessary in order to prevent the sudden release of oxygen and the resulting harmful effects on cells, known as cytotoxicity. Additionally, this approach ensures a consistent and prolonged generation and release of oxygen, which is crucial for supporting cells during the process of angiogenesis. Typically, a range of materials, such as ceramics and polymers, have been employed for the purpose of drug release (Nasiri et al., 2022). In tissue engineering applications, polymers are frequently preferred over ceramics due to their higher biodegradability. Polymers utilized in drug release encompass both synthetic and natural variants, and also their combinations. Presently, a limited number of polymers have been employed for incorporating oxygen sources. These include poly (N-vinylpyrrolidone) (PVP), N-isopropylacrylamide (NIPAAm), poly-ε-caprolactone (PCL), poly (lactide-co-glycolide) (PLGA), and polyurethane (Fan et al., 2018; Mandal et al., 2023). Oxygen-generating sources are commonly combined with carrier polymers, and the resulting mixture experiences various processing methods. These methods are employed to produce diverse forms of oxygen-generating materials. These polymers possess several advantageous features, including established biocompatibility, adjustable biodegradability, and the ability to carry oxygen sources. Moreover, hydrogels, such as alginate and gelatin methacryloyl (GelMA), have been investigated for their potential in 3D regenerative applications (Alemdar et al., 2017; Nejati et al., 2020). These hydrogels not only incorporate sources of oxygen but also offer the ability to encapsulate cells. A recent investigation involved the examination of gellan gum hydrogel combined with CPO. The results demonstrated that this combination was capable of providing oxygen for a duration of 64 h under hypoxic conditions (Newland et al., 2017). A novel biomaterial composed of hyaluronic acid and perfluorocarbon (Oxygel) was also developed. Oxygel is self-healing and shear-thinning, and it can transport substantial oxygen payloads and release them gradually over a period of 90 h (Domingo Lopez et al., 2023).

The selection of materials for use as carriers or for their adsorbing function is mostly determined by the mechanisms of degradation and release time, which are considered to be among the key parameters. One potential approach to controlling the degradation rate and achieving prolonged release of oxygen is to change the hydrophilic properties of polymers. In addition to the investigation of biodegradable polymers, nondegradable polymers like polydimethylsiloxane (PDMS) were also examined. These polymers possess distinct applications or uses in situations where the durability of the device or implant is prioritized (Accolla et al., 2023). Moreover, the current advancements and growing attention towards stimuli-responsive biomaterials present novel opportunities for the regulation of oxygen generation through the use of both local and external stimuli. Oxygen generation can be achieved by utilizing polymers that are responsive to specific triggering factors. These factors can either be local, such as changes in pH or temperature, or respond to external factors like light, magnetic field, or electric field (Wei et al., 2022). In addition to the already employed carriers, alternative biomaterials, such as ceramics or ceramic polymer composites, present promising possibilities for further exploration, potentially expanding the scope of uses for oxygen-generating biomaterials.

### 3.3 Mechanism of oxygen release

When an oxygen source is enclosed, the carrying polymer or ceramic degrades in the presence of water, resulting in the formation of oxygen. During the initial step, a material capable of generating oxygen, such as MPO or CPO, will undergo a reaction with water. This reaction will result in the production of magnesium dihydroxide or calcium dihydroxide in addition to H_2_O_2_. SPO goes through direct decomposition, resulting in the formation of carbonate ions, sodium ions, and H_2_O_2_. Subsequently, the H_2_O_2_ compound gets dissociated, resulting in the formation of oxygen and water. Hence, the incorporation of a catalyst, including catalase and MnO_2_, in the construct proves to be advantageous in facilitating the efficient conversion of H_2_O_2_ into oxygen.

Catalase is an enzymatic protein that is present in the hepatic tissues and bloodstream of humans. Its primary function is to catalyze the decomposition and reduction of H_2_O_2_ into molecular oxygen and water efficiently (Peng et al., 2023). Abdi et al. used a preimmobilized catalase to alginate and successfully encapsulated H_2_O_2_ within PLGA microparticle. This innovative approach allowed for the secure and controlled decomposition of H_2_O_2_. *in vitro* studies have shown that the use of catalase leads to enhanced cell viability (Abdi et al., 2011). Kang et al. conducted a study to investigate the impact of catalase concentration (ranging from 0 to 100 μ/mL) on both the release studies from alginate-based gels and cell viability of human dermal fibroblasts (HDFs). The use of catalase at concentrations of 50 U/mL or higher resulted in a decrease in the amount of H_2_O_2_ produced. This decrease was found to be linked to an extended release of oxygen lasting for 48 h (Kang et al., 2019). Moreover, it was observed that the gradual release of oxygen was not found to have any harmful cytotoxic effects, even when antioxidants were not present (Alemdar et al., 2017). The efficient catalytic properties of manganese dioxide to decompose H_2_O_2_ have garnered significant interest in biofields owing to its favorable biocompatibility, biodegradability, and economical cost (Kim et al., 2019a; Li et al., 2019). To prevent cartilage damage caused by inflammation-induced oxidative stress, polyethylene glycol-modified MnO_2_ nanosheets (NSs) were applied (Kumar et al., 2019). The reduced form of MnO_2_ (Mn^2+^) also played a crucial function in bone metabolism (Hurle et al., 2021; Li et al., 2022), and was required for the synthesis of proteins such alkaline phosphatase (ALP), osteocalcin (OCN), and osteopontin (OPN) in bone tissues (Brodziak-Dopierała et al., 2013; Yu et al., 2016). Studies have indicated that adding Mn2+ to bioactive glass boosts bone growth (Li et al., 2021). The combination of MnO_2_ and CPO has been shown to enhance the dissolved oxygen level to as high as 11 mg/L and to prolong the release of oxygen by at least 75 min. There was a correlation between the MnO_2_ concentration and the oxygen release profile. The more the MnO_2_, the more oxygen is added (Hu et al., 2020). It is evident that the release of oxygen in bursts is a significant factor that can have detrimental effects on cells. Therefore, it is essential to implement a mechanism that prevents burst releases in order to establish a safe platform for oxygen generation.

Oxygen can be liberated from endoperoxides via a novel technique called the RetroDielsAlder reaction. Oxygen is produced when organic compounds like pyridone are heated with endoperoxides (Benz et al., 2013; Changtong et al., 2013). It Covalent interactions allow endoperoxide to be incorporated into the polymeric scaffold, allowing for regulated oxygen release. Photosynthetic algae provided the oxygen by following the mechanism of photosynthetic oxygen synthesis. Other possible include the oxygen diffusion exhibited by microtanks, and microbubbles. Besides these, PFCs follow the mechanism of gas diffusion from emulsions (Agarwal et al., 2021). Various types oxygen generating materials and their associated oxygen release mechanism and kinetics are summarized in Table 1.

**TABLE 1:** Types of oxygen releasing materials and their oxygen-payload capacities, oxygen-release mechanisms, and prevalent applications.

Materials	Preparation methods	Oxygen payload	Mechanisms	Kinetics of oxygen release	Application	Drawbacks	Ref
Solid peroxides	Emulsification, microparticles encapsulation, hydrogel encapsulation, and electrospray	High	Hydrolysis	Hydrolytic decomposition; sustained-release; extended-release over a period of weeks; burst release in the absence of encapsulation	Bone regeneration, vascularization, wound healing, angiogenesis, and osteonecrosis,	Produce ROS, releasing metal cations, increase local pH, and required catalyst to minimize the cytotoxicity of by-products	Lee et al. (2015), Abdullah et al. (2020), Mohseni-Vadeghani et al. (2021), Wang et al. (2021)
PFCs	Emulsification, microparticles encapsulation, hydrogel encapsulation, and grafting	Low	Intermolecular forces	Rapid and then gradual physical diffusion for a period of 48 h; rapid response to ultrasound and temperature for controlled release; recyclable release	Bone regeneration, wound healing, and muscle regeneration	Induces influenza symptoms, fast plasma clearance, insufficient oxygen transport at physiological conditions, and cause vasoconstriction by biding to nitric oxide	Hoganson et al. (2008), Kimelman-Bleich et al. (2009), Wijekoon et al. (2013)
H_2_O_2_	Complexation, microparticles encapsulation, hydrogel encapsulation, and *in situ* generation	High	Disproportionate decomposition	Enzymatic decomposition; sustained-release; extended-release over a period of weeks; burst release in the absence of encapsulation	Muscle tissue, cardiac tissue, and pancreatic tissue engineering	Required catalyst to minimize the cytotoxicity of by-products, induce oxidative damage and cell apoptosis at higher concentration	Abdi et al. (2013), Coronel et al. (2017), Fan et al. (2018)
Hemoglobin	Complexation, microparticles encapsulation, hydrogel encapsulation, electrostatic spraying, molecule modification, and grafting	Lowest	Coordinate bond with ferrous ions (Fe^2+^)	Rapid and then gradual physical diffusion for a period of 24 h; rapid response to temperature and pO_2_ for controlled release; recyclable release	Wound healing, artificial blood and Kidney tissue engineering	Short half-life, produce ROS on oxidation, and cause vasoconstriction by biding to nitric oxide or decomposition	Pusateri et al. (2019), Mahboub et al. (2020), Jebril et al. (2022)
Microalgae	Microparticles encapsulation, hydrogel encapsulation, and 3D printing scaffold	Highest	Photosynthesis	Biochemical reaction, extended-release duration as long as the microalgae is alive, light-responsive controlled and sustained-release	Bone regeneration and wound healing	Applications are restricted because of light exposure needs and likely not immune-compatible	Granito et al. (2017), de Andrade et al. (2022)

## 4 Fabrication of OGBs and bone scaffolds

Biomaterials capable of producing O_2_ have been created through the integration of O_2_ sources into a variety of geometries, including microparticles (Abdi et al., 2013; Mallepally et al., 2014), fibers (Wang et al., 2011; Lu et al., 2019), films (Harrison et al., 2007; Steg et al., 2015) and 3D scaffolds (Nejati et al., 2020). The architecture of scaffolds may have a significant impact on the rate of O_2_ source particle degradation and, consequently, the amount of O_2_ released. For instance, when utilizing extremely porous O_2_ generating materials like nanofiber-based scaffolds, the surface area is very large, and this leads to higher exposure of the material to an aqueous environment, which in turn accelerates material breakdown and, therefore, O_2_ release. Several fabrication techniques have been established to address the existing constraints in regenerative engineering, utilizing modern engineering technology. In the subsequent section, we have examined the most recent advancements in the production of these OGBs as well as their modifications to specifically target the hierarchical structure of the tissue in order to accomplish effective regeneration.

### 4.1 Electro-spraying and electrospinning

**FIGURE 1:**
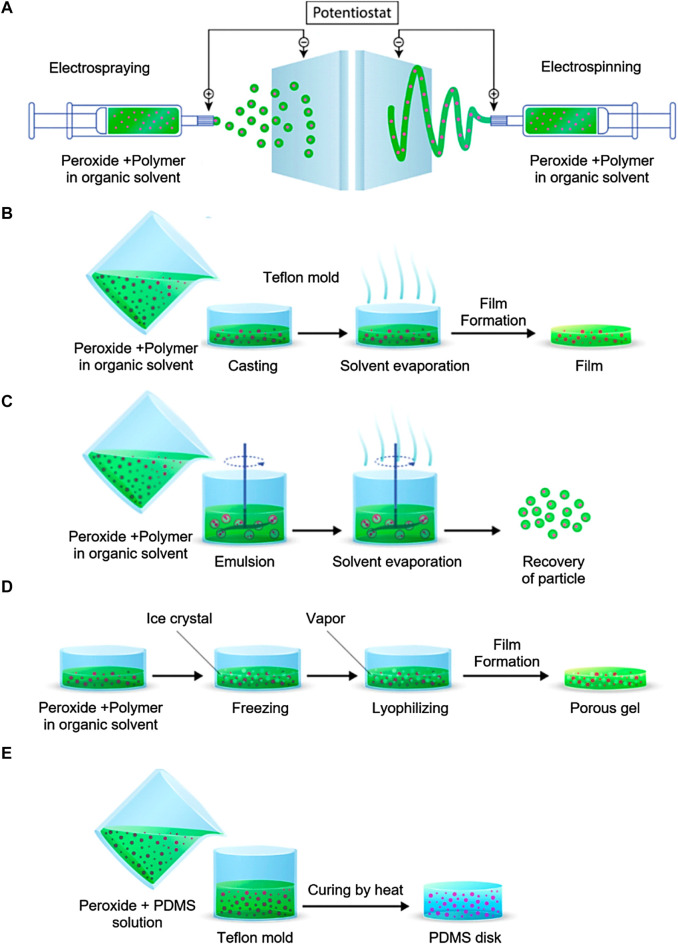
OGBs preparation methods. **(A)** electrospraying and electrospinning method, **(B)** Solvent casting and evaporation method, **(C)** emulsion solvent evaporation method, **(D)** freeze-drying method, **(E)** encapsulation in PDMS. Reproduced with permission from ACS 2019 (Ashammakhi et al., 2019b).

Electrospinning and electrospraying are electrohydrodynamic processes that involve the application of electrically charged polymer solution streams, either single or multiple, to spin or spray in order to generate fibers and particles ranging in size from nanoparticles to microparticles (Figure 1A). (Bhushani and Anandharamakrishnan, 2014). The kinetics of oxygen release can be precisely controlled by encapsulating oxygen-releasing components in nanofibers. A wide variety of synthetic and natural polymers can be treated, and their fiber diameter and orientation can be easily adjusted. Ultrasonic aqueous baths are commonly used to collect the particles or fibers after they have formed and solidified, prior to solvent evaporation. Aleemardani et al. conducted a study wherein they inserted CPO with varying concentrations (ranging from 0.1% to 1%) into silk fibroin to form an electrospun substrate designed for tissue engineering purposes (Aleemardani et al., 2020). The oxygen release of the scaffolds was observed for a period of 13 days. The results demonstrated that the release of the sample containing 0.1% CPO was similar to that of the control scaffolds. However, the release of oxygen dramatically increased for samples containing 0.5% and 1% CPO.

Core-shell oxygen-releasing microparticles can be fabricated using novel methods including coaxial electrospraying. Zhang et al. (Zhang et al., 2020) utilized a coaxial nozzle in conjunction with a single nozzle to fabricate PCL/CPO microparticles with diameters of 26.4 ± 5.1 μm. These microparticles had both single and double walls and were sprayed via coaxial electrospray. In contrast to the single-walled PCL/CPO microparticles that were electrosprayed, the double-walled structure exhibited an enhanced barrier against external water and a decrease in oxygen release. Ma et al. (Ma et al., 2020) recently developed a perfluorotributylamine (PFTBA)-PCL core-shell microfiber system for nerve regeneration by prolonging the oxygen release of the PFTBA via coaxial electrospinning with concentric spinnerets and outer needle diameter 1.2 mm and interior needle diameter 0.3 mm. Core-shell microfibers with diameters of 7.5 ± 2.2 μm and 3.2 ± 2.4 μm were consistently produced by a maximal positive voltage of 16 kV.

CPO, typically used in microscale applications, has been found to have nanoscale properties. CPO nanoparticles were incorporated into PLGA formulation to produce microparticles capable of releasing oxygen using electrospray technique (Khorshidi et al., 2020a). The developed PLGA microparticles exhibited a size range of 4.6–5.3 μm, with the CPO nanoparticles evenly distributed all over the microparticles. The hybrid system demonstrated enhanced viability of mesenchymal stem cells (MSCs) for a longer duration of 14 days. Furthermore, the levels of extracellular matrix (ECM) mineralization and bone-specific markers exhibited an increase, suggesting a favorable impact on the differentiation process towards an osteogenic lineage. The same researchers have also used electrospinning to produce PLA/CPO nanofibrous mats by dispersing nanoscale CPO in a polylactic acid (PLA) solution (Ng et al., 2010). By combining PLA with a more hydrophilic/hydrophobic polymer, adjusting the PLA-to-CPO ratio, and generating surface features on fibers, the degree of oxygenation may be controlled. These matrices effectively promoted MSC culture and differentiation. Microfibers and microparticles with a single phase can be produced via single axial electrospinning or electrospraying. For instance, de Sousa (de Sousa Araújo et al., 2021) used electrospraying at a positive voltage of 12 kV to generate a PCL microparticle loaded with CPO nanoparticles. The hydrogel was utilized to encapsulate the microparticles, which varied in size from 5 to 15 μm, in conjunction with cells to promote accelerated cell division. This resulted in a sustained release of the nanoparticles for a duration of 7 days. At a constant voltage of 15 kV, (Khorshidi et al., 2020a) electrosprayed nano-CPO-loaded PLGA microparticles with an average diameter of 5.3 μm. Even though there was a large range in particle size, the electrosprayed PLGA/CPO microparticles showed a distinctive biconcave disk morphology.

While electrospraying and electrospinning offer many benefits due to their high production and encapsulation rates, one common drawback is the challenge of maintaining precise size. Recent examples of fabricated micro-sized particles show that optimization of experimental settings was done arbitrarily, without a guiding principle. Electrospinning and electrospraying are two procedures where numerous variables, such as voltage, flow rate, and spray height, can be adjusted to achieve optimal results. One of the characteristics that has frequently been necessary for propelling a polymer solution at a consistent velocity is a high voltage within the range of 5 kV–20 kV. However, the ability to achieve such high voltages has been constrained by the limitations of the machinery’s strength and safety considerations. Therefore, advancements in electrodynamic processing with regards to fundamental equipment like the quality of micronozzle array (Zhang et al., 2012) are likely to improve the total production quality of micro-sized oxygen generating carriers. Furthermore, coaxial electrodynamic processes are noteworthy due to their potential for generating microparticles featuring multiple layers (Yoon et al., 2018). This properties-enhancing capability may facilitate the development of novel microparticles having multilayered structures.

### 4.2 Solvent-casting and evaporation

Oxygen source encapsulation can also be achieved through the process of solvent evaporation from an emulsion, which is a widely used technique for fabricating film-based scaffolds. Solvent casting is a conventional fabrication technique employed to establish the shape and pore size of scaffolds. The inclusion of components, such as CPO, can be achieved through their integration into the solubilized carrier matrix and surfactants. After the evaporation of the solvent, the surfactant is eliminated in order to obtain pure particles that are incorporated within the polymer (Figure 1B) (Wang et al., 2020b). Steg et al. reported a study where they used the solvent casting method to make composite films out of polylactic acid (PLA), PLGA, and CPO (Steg et al., 2015). The incorporation of polymers in the composite films resulted in an enhanced rate of oxygen release. The process of hydrolyzing polymers resulted in a decrease in pH levels. Consequently, this led to an increase in the solubility of calcium hydroxide, an intermediate compound. As a result, larger quantities of H_2_O_2_ and oxygen were generated. SPO was encapsulated in a PLGA polymer dissolved in methylene chloride by Harrison et al. Film was demonstrated to produce O_2_ for up to 70 h (Harrison et al., 2007).

Hydrogels with the ability to release oxygen and Mg^2+^ are beneficial for bone regeneration. Seyyed et al. used a solvent casting technique to create hydrogels with MPO-loaded PLGA microparticles, enhancing the release of these substances. The hydrogels were synthesized using methacrylated gelatin and thiolated alginate, and then encapsulated with MPO microparticles. The hydrogels showed favorable cell viability and adhesion characteristics, and the alkaline phosphatase activity of hAMSCs. After 21 days, the hydrogels showed osteogenic differentiation, with the presence of alkaline phosphatase, osteopontin, and osteocalcin, key markers associated with bone formation. Mohseni-Vadeghani et al. developed microparticles capable of generating oxygen by integrating CPO nanoparticles into PLLA microparticles using solvent casting technique (Mohseni-Vadeghani et al., 2021). The microparticles had solid-filled internal structures and were modified with the catalase enzyme to expedite the breakdown of H_2_O_2_ into oxygen. These CPO-loaded PLLA microparticles could release dissolved oxygen and calcium ions for up to 15 days. Moreover, hASCs could adhere to the microparticle surfaces, retaining their characteristic morphology and spreading behavior. The study suggests that oxygen biomaterials can be integrated with injectable micropartilce systems for bone tissue engineering applications.

The solvent casting and evaporation technique represents an easy approach for fabricating films. This method is advantageous for the production of OGBs due to its utilization of organic solvents that do not induce the release of oxygen during the processing stage (Erdem et al., 2022). However, this procedure is time-consuming, hence posing challenges in achieving a homogeneous dispersion of solid peroxide particles within the carrier film. This is primarily due to the tendency of these particles to settle near the bottom of the film. The efficacy of this process may be influenced by various parameters, including the concentration of the polymer, which in turn impacts the thickness of the resultant films. By employing this approach, it is possible to develop structures in a sequential manner, thereby facilitating the development of many layers of uniform compositions. For instance, this technique can be employed to produce solid peroxide particles embedded within a carrier matrix.

### 4.3 Emulsion solvent evaporation

The other approach to encapsulating compounds is the evaporation of the solvent from an emulsion. This technique can be applied to carrier materials that feature both continuous and scattered phases. CPO-loaded particles and the dissolved carrier matrix and surfactant can be combined in this procedure. Figure 1C shows the final product, pure encapsulated particles, after the solvent evaporation and the surfactant removal. Pharmaceuticals are frequently encapsulated using this technique (Iqbal et al., 2015). Zhang et al. employed this approach to produce CPO-gelatin microparticles with the aim of enhancing the process of bone regeneration (Zhang et al., 2021). A gelatin solution was prepared in phosphate-buffered saline, and CPO was distributed into the solution. The solution was combined with Span80, a paraffin-containing substance, and subjected to stirring in order to generate an emulsion. Then, the CPO-gelatin emulsion was cooled to a temperature of 4°C, cross-linked using glutaraldehyde, and agitated to induce the formation of colloidal particles. Finally, the produced microparticles passed through a washing process followed by freeze-drying for a duration of 48 h. The inclusion of gelatin as an encapsulating agent for CPO resulted in a more prolonged and controlled release of oxygen in comparison to the unencapsulated form of CPO. Ng et al. used a double emulsion solvent evaporation process to encapsulate H_2_O_2_ in PLGA microparticles, producing O_2_ producing materials (Ng et al., 2010). A water-oil emulsion was produced in this study by dissolving PLGA in dichloromethane and then emulsifying aqueous H_2_O_2_ in a homogenizer. The resulting water-oil-water emulsion was stabilized with the addition of this mixture to aqueous PVA solution. Following solvent evaporation, microparticles (25–250 μm) were collected, surfactant (PVA) was removed, and they were freeze-dried, resulting in a limited O_2_ generation duration of 5 h. Mallepally et al. reported the encapsulation of H_2_O_2_ in poly (methyl methacrylate) (PMMA). This work emulsified H_2_O_2_ in water and PMMA in acetone and acetonitrile into a mineral oil surfactant. After the solvent evaporation, microparticles (5–30 μm) were separated in a centrifuge capable for providing sustained O_2_ generation for over 24 h (Mallepally et al., 2014). In addition, Steg et al. produced composite microparticles with a poly (trimethylene carbonate) (PTMC) matrix and CPO by means of an oil-in-oil emulsion process. The CPO microparticles were dispersed using a polymer solution (%7 w/v PTMC in acetonitrile), which was then slowly added to a surfactant containing mineral oil. After the acetonitrile evaporated, the microparticles were collected, washed with n-hexane, and finally dried. The obtained microparticles exhibited sustained O_2_ generation that was continued for 20 days without showing any cell toxicity (Steg et al., 2017).

Utilizing this technique to produce microparticles with an encapsulated O_2_ source is beneficial. It cannot, nevertheless, be utilized to generate monodispersed microparticles. The rate of O_2_ release from the final product can be indirectly affected by the type of surfactant, solution, and solvent employed in the emulsion solvent evaporation technique, which controls microparticle size and morphology. The rate of O_2_ release is proportional to the porosity of particles, which in turn is affected by the solvent evaporation rate. Materials that generate oxygen were developed using emulsions of oil in oil and oil in water. Water is utilized in the oil-in-water system not only for emulsion but also for surfactant removal. It is possible that the reaction between peroxide and water during this phase may immediately initiate the degradation of the peroxide. This can be prevented with the use of an oil-in-oil emulsion system (Ashammakhi et al., 2019b).

### 4.4 Gelation

The gelation process relies on the consolidation of a substance using various physical or chemical techniques, such as thermal gelation, polycondensation, copolymerization, and ionic cross-linking (Sun et al., 2021; Hakiki and Arifurrahman, 2023). The process of gelation can be employed to synthesize hydrogels that release oxygen by combining the initial polymer solution with components that generate oxygen. Additional adjustments can be introduced to integrate various components, such as sources that provide oxygen and different combinations thereof, into the existing process. Montesdeoca et al. employed a hydrogel composed of GelMa that is photo-cross-linkable and contains CPO in their study on cartilage tissue engineering (Montesdeoca et al.). GelMa was combined with CPO at concentrations of 0.5%, 1%, and 3% and then solubilized in phosphate-buffered saline. Following this, the Irgacure photo-initiator was introduced, facilitating the cross-linking of the hydrogel through exposure to ultraviolet light. The findings of the study revealed that the initial day’s oxygen release for samples containing 0.5% and 1% CPO was measured at 9.8% and 10.1%, respectively. However, the sample with 3% CPO exhibited significantly higher oxygen release (52.3%), and this sustained release of oxygen persisted for a duration of 2 days. Zhao et al. developed a scaffold that releases oxygen to facilitate bone regeneration. This was achieved by integrating CPO with alginate-polyacrylamide hydrogel (Zhao et al., 2021). The experimental findings demonstrated that hydrogel scaffolds loaded with CPO exhibited a gradual rise in oxygen content over a period of 12 days.

It is possible to further develop this technique to incorporate a wider variety of materials and O_2_ sources. This technique is distinguished by its comparatively quick fabrication of materials that generate oxygen. Nonetheless, the rapid O_2_ emission may also be a limiting factor. In order to extend the duration of release, O_2_ can be incubated in a hydrophobic coating before being combined with the gel.

### 4.5 Freeze-drying

Freeze-drying presents itself as a viable alternative for the encapsulation and localized administration of multiple compounds (Figure 1D). By employing a wide variety of substances, the process of lyophilization uses state-of-the-art advancements to fabricate porous 3D matrices capable of releasing oxygen. The freeze-drying process offers the benefit of employing oxygen sources combined with a solution containing a carrier material. Under reduced-pressure conditions, the solid solvents undergo sublimation, resulting in their efficient removal. The freeze-drying technique demonstrated exceptional efficacy in the fabrication of porous structures comprising a homogenous distribution of solid peroxide particles. However, neither solvent may be used for dissolving the encapsulating compound because only DMSO and water are suitable for the freeze and thaw procedure. Sublimating the solidified solvent after cooling the polymer solution below its freezing point produces a stable structure with connected pores (Valkama et al., 2021; Yuan et al., 2021). The absence of high temperatures that could reduce the activity of biological components is a major benefit of this procedure. Additionally, the pore size can be modulated by adjusting the freezing technique. Antioxidant polyurethane (PU) matrices with different CPO weight percentages were synthesized using freeze-drying in a recent study (Shiekh et al., 2018). After combining CPO with PU in a 5% (w/v) DMSO solution, the resulting combination was frozen and thawed in water or ethanol to form a cryogel. Finally, the cryogels were freeze-dried, resulting in very porous matrices that were unaffected by the varying amounts of CPO used. When CPO was included, oxygen production skyrocketed and stayed high for the next 10 days.

Freeze-drying is an effective technique for making porous scaffolds with evenly dispersed solid peroxide particles. Nevertheless, the freezing and thawing process is limited to the use of water and DMSO alone, hence preventing their ability to dissolve the carrier material.

### 4.6 Polydimethylsiloxane (PDMS) curing

It is possible to encapsulate oxygen sources within hydrophobic PDMS to extend the duration of oxygen release. The experimental procedure involves the combination of oxygen source particles with a PDMS solution, followed by subjecting the resulting mixture to thermal treatment for PDMS polymerization (Figure 1E). Pedraza et al. used PDMS to encapsulate CPO with varying ratios and subjected it to a curing process at a temperature of 40°C for a duration of 24 h (Pedraza et al., 2012). It has been observed that the use of PDMS can effectively prolong the duration of oxygen release to a period exceeding 7 weeks. In a separate study, McQuilling et al. synthesized oxygen-generating films through the process of curing a mixture of SPO and PDMS (McQuilling et al., 2017). Using this technique, the occurrence of burst release was effectively avoided, thereby enabling sustained oxygen release from these films over a duration of 4 days in an *in vitro* setting. Ring scaffolds were also synthesized through the utilization of CPO incorporating PDMS, as demonstrated by Lee et al. The scaffold exhibited sustained oxygen release for a duration exceeding 24 h in an *in vitro* setting (Lee et al., 2018).

While the use of PDMS-based systems exhibits the potential for an elongated release of O_2_, their viability for *in vivo* implementation raises concerns due to their lack of biodegradability. Therefore, these systems would either persist within the organism indefinitely or require surgical intervention for removal (Lee et al., 2018). This technique is advantageous for generating thin structures as water lacks the ability to deeply infiltrate PDMS for facilitating the decomposition of loaded peroxide. In prospective scenarios, the integration of PDMS with diverse biomaterials could yield novel composites exhibiting distinct release characteristics.

### 4.7 Oxygen-carrying microfluidic devices

Microfluidic devices exhibit channel dimensions within the range of 10–1,000 μm, enabling the control of small volumes of fluid. Microfluidic technology is highly advantageous in facilitating the development of physiological microenvironments characterized by 3D designs and precise functionalities at the microscale (Mansoorifar et al., 2021; Sun et al., 2023). Besides that, 3D metrics enable precise control of temporal and spatial parameters within cell culture. When compared to alternative *in vitro* bone models, microfluidic devices demonstrate a lesser requirement for cellular resources and extracellular matrix material (Nasello et al., 2021). They exhibit versatile configurations with different sizes and geometries at the microscale, facilitating the co-culturing of multiple cell types together. Additionally, by housing these cells in distinct compartments, it becomes feasible to observe individual cells separately or investigate cell-cell interactions through the fluidic interconnection. The application of such a design confers several benefits in the context of replicating complex microenvironments and conducting deep studies into the underlying mechanisms of diverse processes, such as bone remodeling (Borciani et al., 2020).

One further characteristic of the microfluidic device is its ability to offer hydrodynamic stimulus, for example, the continual flow of media across a microfluidic channel. The presence of dynamic fluid flow plays a major role in providing the bone cells with the necessary mechanical cues for their growth due to the constant mechanical loading and stimulus experienced by the natural bone (Altmann et al., 2014; Chen et al., 2020). The microfluidic system resembles the circulatory system, facilitating the transport of metabolites, nutrients, and oxygen, which plays a vital role in simulating the metabolic interactions between adjacent tissues and bone. Huang et al. developed a microfluidic microcapsule, consisting of a core of PFC carrying oxygen and a hydrogel shell containing an anticancer drug and sonosensitizer, to enhance chemo-sonodynamic therapy in tumor treatment (Huang et al., 2022). The microcapsules counteract hypoxic microenvironments and increase reactive oxygen species generation, demonstrating potential for synergistic therapeutic approaches in cancer organoid models. The results suggested that microfluidic microcapsules could enhance chemo-sonodynamic therapy in hypoxic tumors. Ochoa et al. successfully designed a cost-efficient and easily transportable system with the capability to deliver oxygen specifically to a localized region of injured tissue while minimizing any potential impact on adjacent healthy tissues (Ochoa et al., 2014). The oxygen delivery system was constructed using PDMS and parchment paper, upon which microfluidic tubes were created. Hydrogen peroxide was employed within the PDMS channels to facilitate the release of oxygen onto the paper substrate. The paper itself contained catalytic areas embedded with manganese dioxide. The microfluidic channels, which incorporated the oxygen-releasing component, exhibited a controlled release of oxygen.

Despite the substantial benefits inherent in this technology, there are a number of challenges that deserve attention and resolution. PDMS is widely recognized as the predominant material chosen as a substrate for constructing microfluidic devices. However, it is widely recognized that PDMS possesses a tendency to absorb small molecules, including pharmaceutical substances, potentially influencing the bioactivity of drugs within microfluidic devices designed for analyzing cellular behavior and therapeutic efficacy (Van Mee et al., 2017). One significant limitation of this technology is the absence of comprehensive inter-organ connections and communication (Shyam et al., 2023).

## 5 Sustained release of oxygen from scaffolds

Although the production of oxygen can lead to the release of potentially toxic H_2_O_2_ and free radicals, it is important to note that oxygen itself can also be harmful to cells if released in abrupt and excessive quantities (Cui et al., 2023). Although oxygen is essential for cellular viability, it must be carefully regulated for four reasons: (1) Cellular oxidative damage due to ROS is a well-documented effect of hyperoxia (Zhou et al., 2014), (2) ROS production and oxygen concentration influence cell differentiation (Atashi et al., 2015), (3) ROS facilitates the natural inflammatory response (Mittal et al., 2014), and (4) Vascular infiltration is triggered by moderate hypoxia (Lam and Sefton, 2015; Lewis et al., 2015). To ensure that there is sufficient oxygen to maintain cell viability without impeding vascularization, differentiation, tissue damage from inflammation, or the release of an excess of oxygen, it is vital to comprehend the oxygen requirements of specific tissues and the rate of vascularization into those tissues. Furthermore, hyperoxia can produce major hemodynamic changes (Cornet et al., 2013; Sjöberg and Singer, 2013) and has been shown to cause vasoconstriction in some vascular systems (coronary, cerebral, skeletal muscle, and retinal) (Messina et al., 1994; Kiss et al., 2002; Floyd et al., 2003; McNulty et al., 2007), but not all (renal, mesenteric) (Lang and Johnson, 1988; Sha et al., 1998), vascular beds. This vasoconstriction appears to occur primarily at the microvascular level, as large conduit arteries diameters remain constant (McNulty et al., 2007; Farquhar et al., 2009). Loss of functional capillary density and decreased organ perfusion may result from microcirculatory heterogeneity (Kamler et al., 2004; Cornet et al., 2013). Conversely, in the event of a slow release of oxygen, there exists an insufficiency in the transportation of this vital element to the cellular entities within the engineered tissue, thereby preventing the sustenance of optimal cellular functionality. Therefore, the regulation of oxygen release kinetics is of utmost importance. Various strategies have been explored to regulate the discharge of oxygen from oxygen-generating materials, with particular emphasis on the use of nanostructured materials and polymeric carriers as the most productive approach for achieving controlled release of the encapsulated oxygen (Suvarnapathaki et al., 2022; Augustine et al., 2023). The initial approaches employed to accomplish this objective involved the utilization of microparticles composed of hydrophobic polymers as carriers for oxygen-releasing substances.

The use of hydrophobic materials for the encapsulation of oxygen-generating compounds leads to a reduction in the rate of oxygen release. This is attributed to the hindered passage of water through the hydrophobic substance (Suvarnapathaki et al., 2019). In contrast, it has been observed that hydrophilic carriers that contain oxygen-generating compounds exhibit a high affinity for water, leading to the rapid adsorption of water molecules. This phenomenon promotes the decomposition of solid peroxide particles, resulting in an accelerated production of oxygen (Alemdar et al., 2017). Numerous studies have demonstrated the effective use of polymers with inherent stability in physiological environments, including PLGA, PLA, PCL, and other biodegradable polymers, for the purpose of delivering oxygen over extended periods from scaffolds composed of biomaterials (Nejati et al., 2021; Suvarnapathaki et al., 2021). A recent study reported PCL microparticles that were encapsulated with CPO, with the aim of exploring their possible applications in the field of bone regeneration (Suvarnapathaki et al., 2022). The introduction of microparticles into GelMA-based scaffolds resulted in a substantial enhancement in both cell survival and proliferation. It is interesting that microparticles containing CPO demonstrated reduced cytotoxicity due to the controlled and slow oxygen release for a minimum duration of 2 weeks in a hypoxic environment. The use of perfluorooctane emulsion oxygen carrier-loaded hollow microparticles resulted in the sustained release of oxygen and the prevention of cell necrosis for a duration of about 2 weeks inside a hypoxic environment. This effect persisted until the formation of new blood vessels occurred within the 3D construct (Lee et al., 2015). Moreover, the use of microparticle fabrication methods such as electrospraying has been reported for the development of multi-walled, persistent, and controlled oxygen delivery systems (Zhang et al., 2020). The lifespan of cells cultured with double-walled microparticles loaded with CPO was shown to be higher compared to cells cultured with single-walled microparticles. Probably, this phenomenon occurred due to the progressive release of oxygen from the double-walled particles in comparison to the single-walled particles. Chengqiang et al. fabricated 3D-printed scaffolds with CPO-gelatin-loaded microparticles for the purpose of mending the osteonecrosis-induced impairment in the femoral head (Wang et al., 2021). The scaffolds, which encompassed the bone marrow mesenchymal stem cells (BMSCs), were meticulously introduced into the central depression region within the femoral head of the osteonecrosis rabbit model. The findings revealed that the CPO-gelatin-loaded microparticles exhibited a sustained release of oxygen over a period of 19 days. Further analysis has indicated that the incorporation of microparticles into the scaffolds could decrease local cell apoptosis and promote the viability of the transplanted cells within the receiving organism.

Controlled oxygen delivery methods exhibit considerable potential as effective procedures for producing substantial tissue engineered-structures and potentially viable tissue constructs of organ size with clinical significance. Furthermore, it is possible to develop OGBs that effectively regulate the kinetics of oxygen release. Research findings have demonstrated that, in comparison to other solid peroxides, MPO exhibits a slower rate of oxygen formation owing to its relatively reduced solubility. The equilibrium coefficients for the reactions of CPO and MPO with water are reported as 9.8 × 10^−9^ and 1.8 × 10^−11^, respectively. These values indicate that MPO exhibits a lower solubility compared to CPO, consequently resulting in a slower reaction rate when compared to CPO (Waite et al., 1999).

## 6 Applications of OGBs in bone regeneration

Although bones have an inherent ability to undergo repair following injury, the healing process might be hindered in certain complex clinical scenarios, such as those with significant loss of bone mass or defective regenerative mechanisms (Tang et al., 2020). A major hurdle encountered in the field of regenerative engineering is the reconstruction of bone defects that are of critical size. This challenge arises from the restricted migration and viability of cells within the inner regions of the expansive matrix. The occurrence of bone injury is accompanied by concurrent damage to the intrinsic vascular network. Despite the inherent regeneration capabilities of bone, osteonecrosis frequently arises as a consequence of diminished blood flow. The occurrence of ischemia is a significant challenge during engraftment procedures in areas characterized by inadequate vascularization. During bone regeneration and osseointegration, it is observed that when the matrices exceed the diffusional limits of oxygen and nutrients, the interior donor cells experience restricted access to nourishment and an accumulation of waste products. As a result, the formation of a necrotic core occurs, leading to inhibition of osseointegration and bone regeneration (Otsuka et al., 2022). Following the transfusion of blood, bone grafting treatments are the second most frequently performed tissue transplantation procedure (Hosseini and Laurencin, 2022). Globally, the number of surgical grafting procedures exceeds two million, resulting in an estimated annual expenditure of over $2.2 billion. At present, there is a lack of established methodologies for the regeneration of damaged tissue. Autologous bone grafts are considered the preferred treatment option due to their inherent ability to facilitate direct bonding, provide mechanical support for bone formation (osteoconductivity), generate bone (osteogenicity), and stimulate the differentiation of local stem cells into osteogenic cells (He et al., 2010).

The engineering of barrier membranes that possess optimal porosity and biological activity is important for facilitating controlled bone formation in orthopedic applications (Wu et al., 2019). To facilitate the adherence and growth of bone cells and promote bone formation, it is imperative for a bone regenerative scaffold to possess certain characteristics, including osteoconductivity, porosity, and biodegradability (Raina et al., 2019). The predominant approach to employing OGBs for bone regeneration engineering involves the use of natural or synthetic biomaterials to produce a matrix for tissue development. Even though the properties of polymers can be tailored in multiple ways, such as surface properties, rigidity, mechanical properties, biocompatibility, and biodegradability, to customize biomatrix design for specific tissue sites. However, there is still a critical knowledge gap when it comes to figuring out the right material combinations to improve the angiogenic and osteoblastic differentiation of stem cells to promote bone tissue regeneration. The provision of a consistent oxygen supply to hypoxic tissues can result in a delay in the initiation of necrosis, hence promoting extended tissue viability and improved wound healing. But the ability to effectively control the rapid release of ions is of utmost importance to prevent any potential toxicity and subsequent cellular death. In order to address this particular difficulty, a range of methodologies were utilized to effectively incorporate CPO nanoparticles within polymers and produce grafts capable of releasing oxygen. In a recent study, nanoscale CPO was dispersed in a PLA solution to electrospun PLA/CPO nanofibrous mats. By adjusting the proportion of PLA to CPO, introducing hydrophobic or hydrophilic polymers, and implementing surface patterning on the fibers, the oxygenation levels were enhanced, leading to effective promotion of MSCs proliferation and differentiation. The study aimed to evaluate the efficacy of pure and CPO-coated biphasic calcium phosphate (BCP) constructions in promoting healing in rabbits’ radial fractures (Khorshidi et al., 2020b).

**FIGURE 2:**
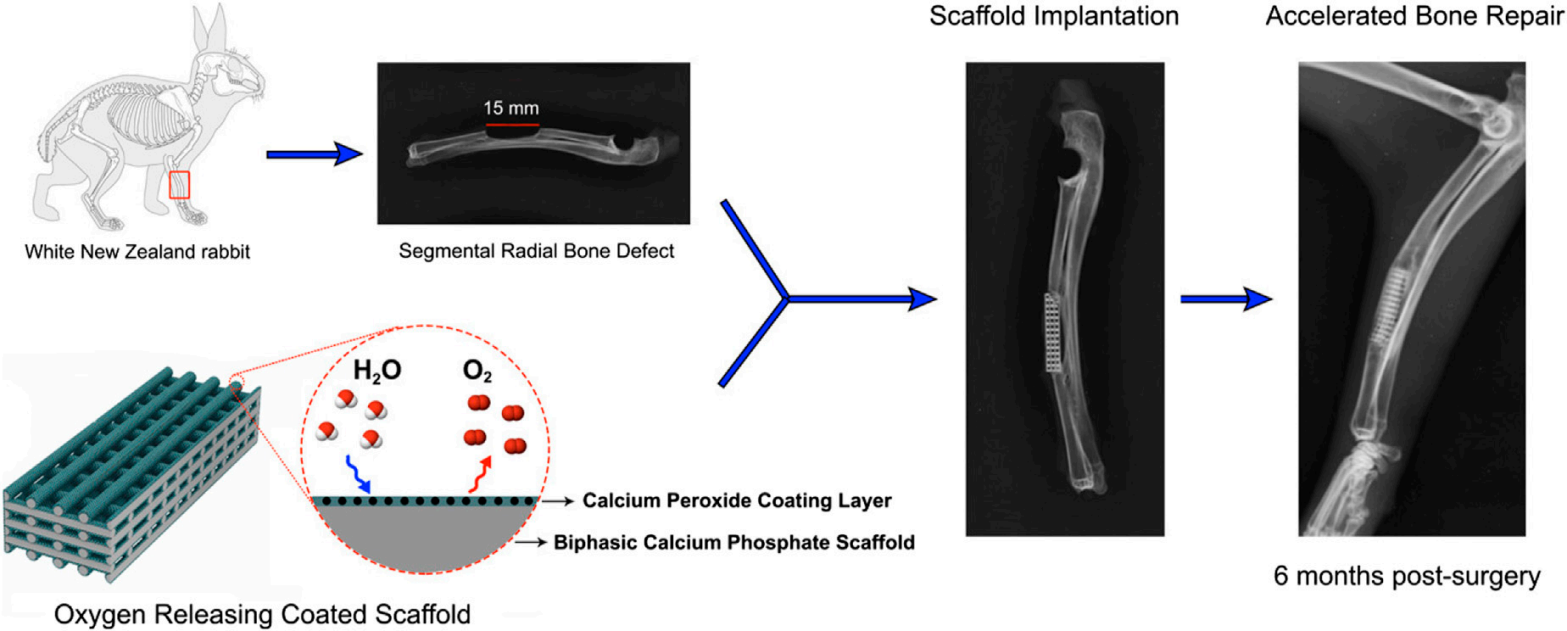
Oxygen-generating coating of the BCP scaffold accelerated the bone fracture healing *in vivo*. Reproduced with permission from ACS 2020 (Touri et al., 2020).

Besides these, BCP scaffolds were produced via robocasting, an additive manufacturing process. As an oxygen-releasing agent, CPO particles were subsequently deposited onto the BCP scaffolds (Touri et al., 2020). In rabbits, segmental radial defects measuring 15 mm in diameter were introduced. In the defects, uncoated and CPO-coated BCP scaffolds were implanted. There was no implantation in the “empty” (control) group. Radiographic evidence suggests that CPO-coated BCP scaffolds, in comparison to their uncoated counterparts, promote more rapid bone growth at the implant/host bone interface and within the scaffolds’ internal pores. New bone formation was approximately twice as abundant in the CPO-coated scaffold compared to the uncoated scaffold, as shown by histomorphometry analysis. Osteogenic markers, namely, osteonectin and octeocalcin, were observed to be overexpressed in defects treated with coated scaffolds 6 months after surgery, as determined by immunofluorescence staining. This group exhibited superior bone mineralization and osteogenic differentiation in comparison to the uncoated scaffold group (Figure 2). This system of oxygen-generating coating/scaffold may be a prospective for rapid healing of bone defects, as the results suggest an enhanced ability of bone repair for CPO-coated BCP scaffolds implanted in the segmental defect of rabbit radius.

Hydroxyapatite (HAp) is extensively employed as a biomineral in many orthopedic interventions. HAp is widely recognized and valued for its osteoinductivity, osteoconductivity, biocompatibility, and bioactivity, making it a crucial inorganic component of bone construction (Mohseni et al., 2014). 3D-printed scaffolds composed of tricalcium phosphate and HAp were fabricated with PCL-CPO for bone regenerative applications (Touri et al., 2018). In the initial stages following implantation, when oxygen levels are low, the use of hydrophobic PCL is implemented to retard the decomposition of CPO and sustain the release of oxygen. During the 10-day experimental period, the production of oxygen was consistently sustained by the encapsulated CPO content. In addition, the scaffolds were found to enhance the cell survival, proliferation, and metabolic activity of osteoblasts. The study found that scaffolds coated with 3% CPO exhibited the highest levels of cell proliferation and vitality in comparison to both uncoated CPO scaffolds and other types of scaffolds.

**FIGURE 3:**
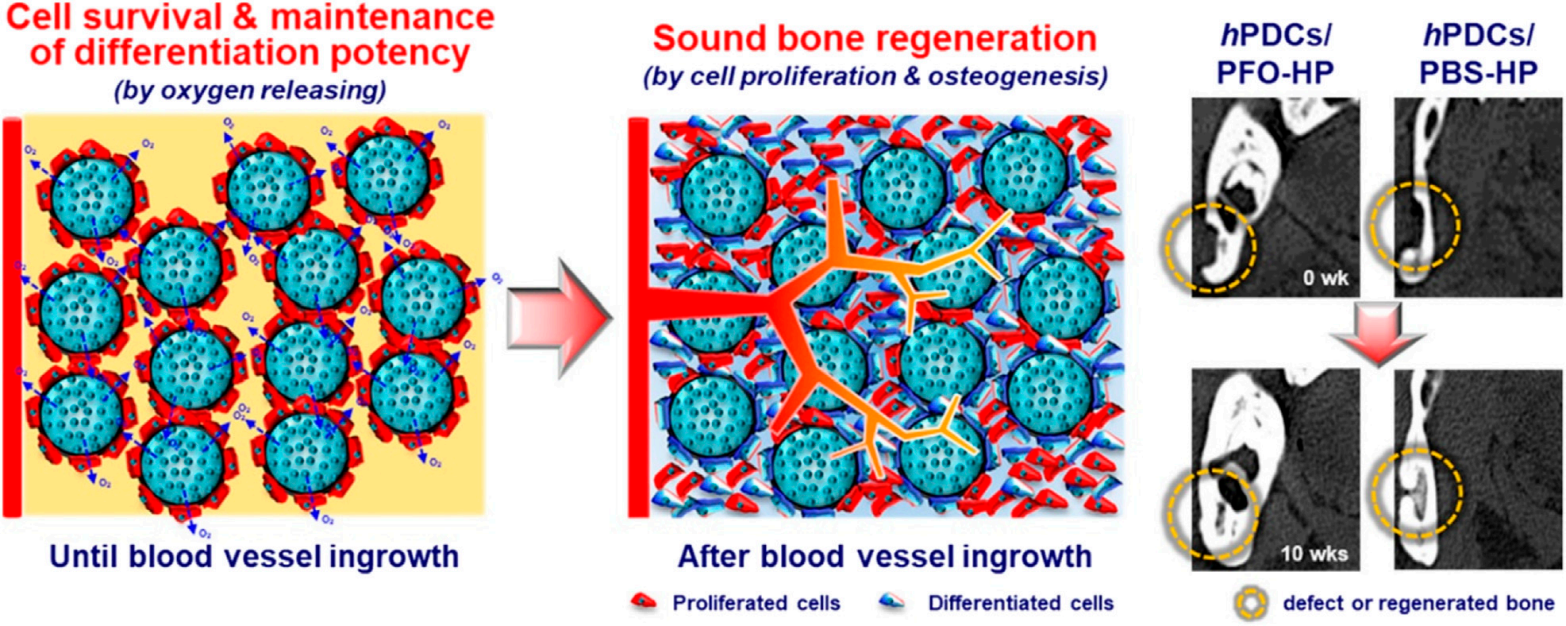
The release of oxygen by PFO-HPs permits cells to survive and maintain their differentiation potential during hypoxia, resulting in bone repair in bony defects. Reproduced with permission from ACS 2019 (Kim et al., 2019b).

A range of OGBs have exhibited the ability to substitute vasculature; however, they may also operate synergistically to ensure timely and efficient delivery to tissues. The use of oxygen-generating matrices that possess predictable oxygen release kinetics and modular material features has the potential to enhance the effectiveness of extended bone tissue implant therapies, which commonly involve a hypoxic environment. To achieve improved bone regeneration in a 3D-tissue construct, Suvarnapathaki et al. used PCL microparticles containing emulsified CPO to strengthen the hydrogel scaffold (Suvarnapathaki et al., 2022). Consistent metabolic activity, tissue viability, and osteogenic differentiation were observed for a period of 2 weeks in both *in vitro* and *in vivo* experiments. The experimental findings revealed the ability of scaffolds to sustain bone volumes of 70 mm^3^ and achieve a regeneration rate over 90% in critical-size cranial fractures. Furthermore, they employed tartrate-resistant acid phosphatase (TRAP) and VEGF staining techniques to validate the outcomes of bone remodeling and vascularization *in vivo*. Recently, there has been a study conducted to test the efficacy of tissue regeneration in mandibular fractures in miniature pig models by evaluating oxygen carriers for *in vivo* applications. This study involved the use of perfluorooctane-loaded hollow microparticles (PFO-HPs) that were seeded with human periosteal-derived cells (Kim H. Y. et al., 2019). In addition, they established that the number of cells initially implanted on PFO-HPs *in vitro* is maintained and that the PFO-HPs supplied adequate oxygen to the surrounding cells during 10 days of hypoxia. Human periosteal-derived cells (*h*PDCs) implanted on PFO-HPs demonstrated a 14-day survival period prior to *in vivo* blood vessel penetration (Figure 3). The findings of this study indicate that the combined use of oxygen-releasing microparticles and cells exhibited a synergistic effect in facilitating the process of bone repair.

In a separate investigation, novel microparticles coated with catalase were developed with the ability to release oxygen in response to the oxygen level in the environment; that is, they released oxygen at faster rates at lower oxygen levels and at a slower rate at higher oxygen levels. The oxygen-responsive shell of the microparticles comprised 2-nitroimidazole, the hydrophilic nature of which increases with a decrease in the oxygen level in the surrounding environment. This resulted in an increased hydrophilic nature and degradation rate of the its shell, which accelerated the release of oxygen. Additionally, the surface of the shell was conjugated with catalase, enabling the microparticles to release molecular oxygen directly. Under hypoxic conditions, the released oxygen substantially increased the survival of MSCs without inducing ROS production. The proliferation, survival, and paracrine effects of MSCs were all improved by co-delivery of MSCs and microparticles to the ischemic limbs of mice. Additionally, it restored blood flow, accelerated angiogenesis, and stimulated skeletal muscle regeneration without inducing tissue inflammation (Guan et al., 2021).

## 7 Challenges

Contemporary approaches to fracture fixation have attained an elevated level of technology that guarantees the methodological reliability of surgical procedures and, on an international scale, a superior standard of medical care. Despite this, there are still a significant number of unresolved clinical issues, comprising 10%–20% of delayed or nonunion cases despite the implementation of innovative treatment approaches. Significant obstacles to effective bone healing include bone loss, defects, inadequate vascularization, soft-tissue damage, insufficient mechanical stability, infections, and tumors (Winkler et al., 2018). They hinder or completely prevent clinical recovery. Presently, the application of oxygen generating biomaterials has been observed to be relatively restricted in scope, primarily due to the inherent complexities associated with achieving efficient peroxide dispersion, offering desirable degradation processes ensuring controlled and continuous release, and compatibility with cellular components. The adjustment of decomposition rates of carrier biomaterials and oxygen release profiles is necessary in accordance with the specific application requirements. The chosen methodology should effectively reduce the possibility of premature burst release while ensuring sustained release and cellular support. It is imperative, however, to minimize the production of reactive oxygen species (ROS) (Pedraza et al., 2012; Shiekh et al., 2018). One potential approach involves employing a composite of biomaterials, whereby several layers of carrier biomaterials are coated with distinct coatings that possess tunable degrading properties. Several OGBs have been discussed that exhibited the prominent efficacy towards bone regeneration but various challenges are still present for such carrier systems. For instance, when the PFC droplets enter the bloodstream, they face a number of obstacles. The microvascular system can be obstructed by microparticles greater than 0.5 μm in diameter, leading to a microembolism, hence the maximum particle dimension should be less than 0.5 μm. Furthermore, increased particulate size decreases the capacity for oxygen transport as a result of reduced diffusivity. PFC-based oxygen carriers have decreased popularity and have been withdrawn from human clinical trials due to this purported hazard (Hosseini et al., 2023). Hyperbaric oxygen can be loaded into microtanks, also known as hollow microparticles. However, the amount of oxygen that can be delivered and the duration of the oxygen release are two of the main challenges of using microtanks as oxygen-releasing materials for tissue regeneration. The PCL microtanks greatly improved *h*ADC-mediated bone tissue regeneration, as reported by Farris *et al.*, however the oxygen delivery was only effective for 8 h (Agarwal et al., 2021).

The integration of 3D bioprinting with microfluidic chips has the potential to facilitate the advancement of materials with enhanced control and uniformity in oxygen delivery rates. The incorporation of remotely controlled devices and stimulus-responsive biomaterials has the potential to be effective in the implementation of oxygen release systems that can be activated as required (Wei et al., 2022; Shi et al., 2023). Although microfluidic-based scaffolding approaches hold promise, certain microfabrication techniques for recreating capillary-like structures at a smaller scale still lack optimal resolution (Ferreira et al., 2018; Suvarnapathaki et al., 2018). Furthermore, it is crucial to balance complexity and functionality of vascular networks when designing such systems. Additionally, growth factors and physiologic fluid flow modeling should not be neglected, nor should multicellular interactions and elements of functional vasculature (e.g., basement membrane, supporting cells) be omitted (Lee et al., 2018). The timely removal of on-chipengineered pre-vascularized microtissues from microfluidic platforms may pose a significant challenge, especially when large-scale parallelization is necessary for the ultimate application. Researchers have investigated various alternatives in an effort to ensure greater control over microtissue oxygenation and *in vivo* integration into pre-existing or newly formed vascular networks, after realizing the inherent limitations of these methods. The use of intelligent materials, namely, those capable of being activated by fluctuations in local pH levels, holds potential for the implementation of oxygen generation systems. For instance, a correlation has been observed between reduced perfusion, ischemia, and decreased pH levels in tissues. This relationship can be used as a stimulus for the release of oxygen from pH-responsive biomaterials (Xie et al., 2022).

Further development of *in vivo* models is necessary to enhance the comprehension of the dynamics of oxygen therapy systems. Sensors have the potential to be employed for the purpose of monitoring physiological responses *in vivo* during the course of therapy as well as determining the profiles of oxygen release (Altyar et al., 2023). Certain cells have a greater demand for oxygen supply, requiring higher levels of oxygen delivery. However, an excessive amount of oxygen might be harmful to other cells. Therefore, it is crucial to establish and monitor the required oxygen delivery specifications and focused localized release.

Efforts have been made to advance the functionalization and/or formulation of specific biomaterials with the purpose of delivering oxygen in a controlled and localized manner, for instance, through injection (Augustine and Camci-Unal, 2023; Norahan et al., 2023). However, the scope of these investigations is slightly limited. Moreover, there is an urgent desire for the advancement of multifunctional biomaterials to ensure the precise delivery of an optimal quantity of oxygen to specific sites for a certain duration while maintaining control and sustainability. These biomaterials should be designed to meet the requirements of clinical applications. The progress made in the research and development of innovative biomaterials that generate oxygen will contribute to addressing many challenges related to regenerative treatments. Further development is required to enhance the scalability of the process in order to facilitate large-scale industrial production. The process of obtaining regulatory permission for active agent-generating biomaterials poses a significant barrier, sometimes resulting in longer approval timelines compared to nondrug-releasing biomaterials and devices. The potential outcome of this situation is a potential delay in the translation of research findings into therapeutic applications. Sufficient data exists to support the advantageous nature of oxygen generating biomaterials. However, it is essential to conduct extensive *in vivo* investigations in order to obtain comprehensive insights and draw definitive conclusions that can facilitate the progression of clinical applications.

## 8 Conclusion and future perspectives

Recent progress in the fields of regenerative engineering and nanobiotechnology has facilitated the activation of the body’s own regenerative processes in the specific environment of healing. This has led to the development of a number of therapeutic implants that are specifically designed for the requirements of damaged organs, with the aim of improving their functionality. Materials science has played a crucial role in advancing the practical application of many regeneration techniques within the field of regenerative medicine. The primary objective of this interdisciplinary discipline is to fabricate biomaterial scaffolds that possess a wide range of topological, compositional, structural, physiochemical and biological attributes. These attributes are carefully developed to respond to various therapeutic objectives. Oxygen generating biomaterials are integrated into the designed matrices as a component that, upon disintegration, emits oxygen. This process serves to mitigate cell death caused by hypoxia, promote bone regeneration, and promote vascularization.

The adoption of many modern technologies, such as scaffold fabrication techniques and the expansion of oxygen generating biomaterials, has been employed in the development of breathing frameworks for the purpose of supplying oxygen in bone regeneration. This application of emerging technologies has contributed to the advancement of the field, bringing it closer to potential clinical implementation. Simultaneously, the advancement of oxygen generating biomaterials has the capacity to greatly enhance the efficacy of bone regeneration.

Given the swift growth and development of studies regarding the use of OGBs in the context of bone regeneration, there are a number of crucial elements that deserve consideration. The most difficult component of the process lies in attaining a harmonious and prolonged release of oxygen, which guarantees the survival of cells and mitigates the risk of abrupt oxygen release that could lead to cell damage. The examination of biomaterials’ functioning and biocompatibility is being conducted by researchers in order to gain a full understanding of how these materials function in target tissues. In addition, researchers are also investigating and advancing the development of novel oxygen generating biomaterials, as well as exploring new techniques for scaffold production, including 3D bioprinting and microfluidics. In light of the expected path of the field, various methodologies, including machine learning and computational modeling, can be utilized to develop a matrix that accurately simulates the hierarchical characteristics of impaired tissue. This matrix can then be optimized to determine the necessary variables for an effective approach to tissue regeneration. Nevertheless, there has been substantial progress in the advancement of OGBs in recent years, leading to the expectation of their future use in clinical settings.
